# A review of the genus *Monema* Walker in China (Lepidoptera, Limacodidae)

**DOI:** 10.3897/zookeys.306.5216

**Published:** 2013-06-03

**Authors:** Zhaohui Pan, Chaodong Zhu, Chunsheng Wu

**Affiliations:** 1 Institute of Plateau Ecology, Agriculture and Animal Husbandry College of Tibet University, Linzhi 860000, P.R. China; 2 Key Laboratory of Zoological Systematics and Evolution, Institute of Zoology, Chinese Academy of Sciences, Beijing 100101, P.R. China

**Keywords:** Lepidoptera, Limacodidae, *Monema*, new species, China

## Abstract

Four species and one subspecies of the genus *Monema* Walker, 1855 are recognized from China, in which *Monema tanaognatha* Wu & Pan **sp. n.** is described as new, *Monema coralina* Dudgeon, 1895 and *Monema meyi* Solovyev & Witt, 2009 are newly recorded for China. The female of *Monema meyi* is reported for the first time. *Monema nigrans* de Joannis, 1901 and *Monema melli* Hering, 1931 are synonymized with *Monema flavescens* Walker, 1855. *Cnidocampa rubriceps* Matsumura, 1931 is regarded here as a subspecies of *Monema flavescens* Walker, 1855. The photographs of moths and their genitalia are given. A key to the species of the genus is provided.

## Introduction

The genus *Monema* was erected by Walker in 1855, based on the type species, *Monema flavescens* Walker, 1855.

Prior to the present study the genus included the following species: *Monema flavescens* Walker, 1855, *Monema nigrans* de Joannis, 1901, *Monema coralina* Dudgeon, 1895, *Monema rubriceps* (Matsumura, 1931), *Monema melli* Hering, 1931 and *Monema meyi* Solovyev & Witt, 2009 and it is distributed in Nepal, Bhutan, China, Far East of Russia, Korea, Japan, and northern Vietnam (Hering 1913, 1933; Solovyev 2008; Solovyev and Witt 2009; Yoshimoto 1993, 1994). The diagnosis of the genus is given below.

In China three taxa have been recorded (Cai 1981; Inoue 1992; Wu 2005, 2012; Wu and Fang 2010) up to now. In this paper, four species are reported for China, including one species described as new to science and two species newly recorded for China. *Monema nigrans* and *Monema melli* are synonymized with *Monema flavescens*. *Cnidocampa rubriceps* is regarded here as a subspecies of *Monema flavescens*.

## Materials and methods

Material examined for this study originates from the insect collections of the Institute of Zoology, Chinese Academy of Sciences (IZCAS), Beijing, P. R. China. All types of new species are deposited in IZCAS

Photographs of moths and their genitalia were captured using Canon-EOS-7D with the help of micro-lens. Standard methods of dissection and mounting in Euparal follow Holloway et al. (1987).

## Systematics

### Monema

Walker, 1855

http://species-id.net/wiki/Monema

Monema Walker, 1855: 1102,1112. Type species: *Monema flavescens* Walker, 1855: 1112, by monotypy.
Cnidocampa Dyar, 1905: 952. An unnecessary replacement name for *Monema* Walker, 1855, [not preoccupied by the plant genus *Monema* Greville, 1827].

#### Description.

Moths medium-sized, body yellowish. Male antennae filiform and thicker than in female. Labial palpi extremely long, more than three times eye diameter. Forewings with R_3_ + R_4_ stalked from R_5_ and R_2_ stalked with their stem. Hindwings with M_1_ and Rs stalked. Forewings with two narrow brown fasciae running from apex to 3/4 and 1/3 of inner margin respectively; basal part of forewing bordered by proximal fascia yellow, rest brown. Hind tibiae with two pairs of spurs.

Male genitalia: tegumen narrow; uncus narrow and long, usually with short ventral process at apex; gnathos narrow and long, nearly as long as uncus; juxta with lateral elongate process or spines; valva elongate, with apically saccular process; saccus usually long and large, more than half of valva’s width.

Female genitalia: posterior apophysis long, anterior apophysis very short and less than the half length of posterior one; sclerotized exterior flap at posterior margin of ostium bearing minute hair; ductus bursae very long, more than the half length of the abdomen, base narrow and straight, apical part wider and spiraled; corpus bursae ovate, large; a pair of signa trigonal, bearing spines.

The larva belongs to the nettle-type, and is known only for *Monema flavescens*. Its final instar larvae are 19–25mm in length. Head yellowish brown. Thorax yellowish green. Dorsum with a large purple-brown spot shaped as a dumbbell. Subdorsal scoli on T2-A9 and lateral scoli on T2, T3 and A2-8 (Fig. 22) (Long et al. 2008).

The cocoon of *Monema flavescens* Walker is very hard and shaped as a sparrow-egg. It is white, with longitudinal brown stripes (Fig. 23).

The genus is related to *Hyphorma* Walker, 1865, but differs from the latter by the shorter terminal segment of the labial palpi and the stalked R_2_ and R_3-5_ in the forewings. *Scopelodes* Westwood, 1841 and *Phocoderma* Butler, 1886 also have very long palpi, but the absence of a tuft of hair in 2nd or 3rd segments distinguish them from *Monema* (Hering 1931).

##### Key to the species and subspecies

**Table:** 

1	Wings mostly pale black, brown or pale reddish	2
–	Wings mostly yellow or yellowish brown	3
2	Wings mostly pale reddish	*Monema coralina*
–	Wings mostly brown to pale black	*Monema flavescens flavescens* (Black form)
3	Frons red	*Monema flavescens rubriceps*
–	Frons yellow	4
4	Saccus long, aedeagus straight	5
–	Saccus short and wide, aedeagus S-shaped	*Monema meyi*
5	Gnathos narrow and long; juxta long, ending in a tuft of long spines each side	*Monema tanaognatha* sp. n.
–	Gnathos short; juxta short, ending in 1-3 long spines each side	*Monema flavescens flavescens*

### Monema flavescens

Walker, 1855

http://species-id.net/wiki/Monema_flavescens

Figs 5, 6 , 10, 12 , 13, 14, 15, 16, 17 , 22, 23

Monema flavescens Walker, 1855: 1112, fig. 1c. Type locality: North China.
Miresa flavescens (Walker): Seitz, 1913: 344, fig. 50c.
Cnidocampa flavescens (Walker): Cai, 1981: 99.
Cnidocampa johanibergmani Bryk, 1948: 219.
Monema melli Hering, 1931: 691, fig. 87i. Type locality: Guangdong, China. syn. n.
Monema flavescens var. *nigrans* de Joannis, 1901: 251.
Monema nigrans de Joannis: Solovyev and Witt 2009: 108.

#### Description.

Wing expanse 30–32 mm in male, 35–39 mm in female. In male genitalia, the juxta is short and ends in 1-3 long spines each side. In female genitalia, the sclerotized base of ductus bursae is diagnostic.

**Figures 1–6.**
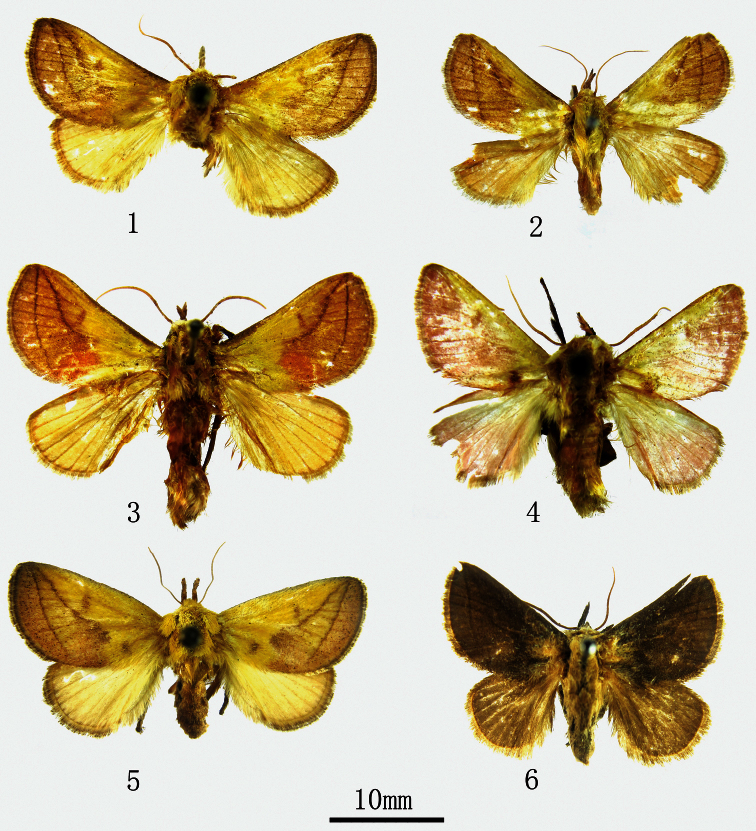
Adults. **1** *Monema tanaognatha* Wu & Pan, sp. n., holotype (Kunming, male) **2** *Monema tanaognatha* sp. n., paratype (Kunming, female) **3** *Monema meyi* Solovyev & Witt (Hunan, male) **4** *Monema coralina* Dudgeon (Yunnan, male) **5** *Monema flavescens flavescens* Walker (Beijing, female) **6** *Monema flavescens flavescens* Walker (Black form, Shanghai, male).

**Figures 7–12.**
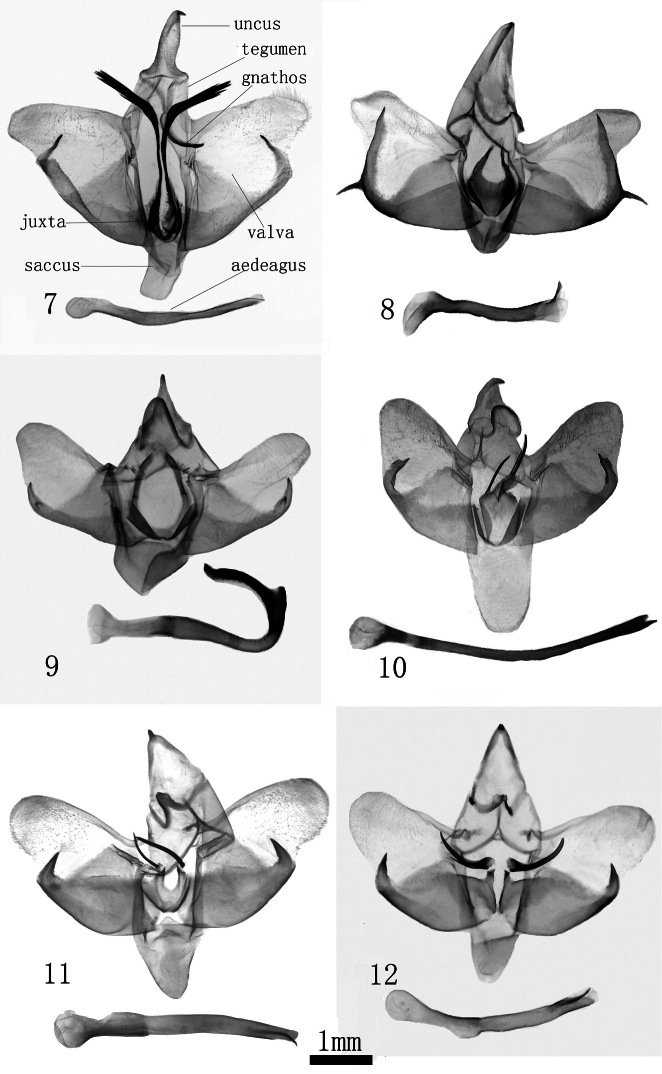
Male genitalia. **7** *Monema tanaognatha* Wu & Pan, sp. n. holotype (gen. slide WU0156) **8** *Monema coralina* Dudgeon (gen. slide L06051) **9** *Monema meyi* Solovyev &Witt (gen. slide WU0121) **10** *Monema flavescens rubriceps* (Matsumura) (16058 MWM GS Taiwan) **11** *Monema melli* Hering, holotype (TYPE ZHUB GU2) **12** *Monema flavescens flavescens* (Black form, gen. slide L06052).

**Figures 13–17.**
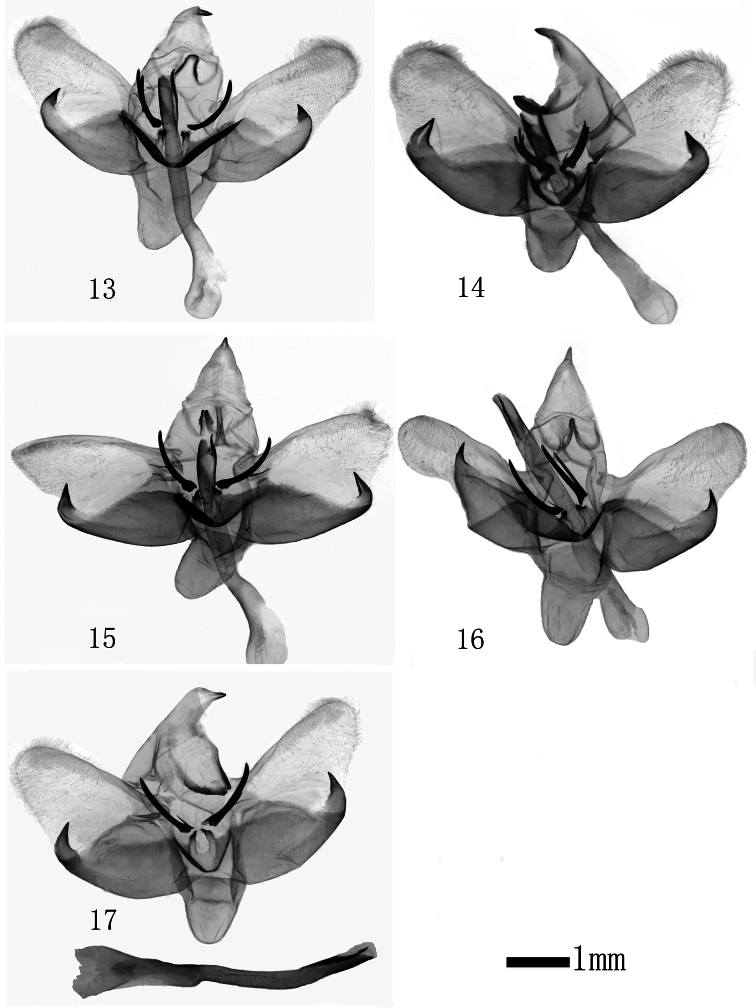
Male genitalia of *Monema flavescens flavescens* Walker. **13** Shennongjia, Hubei (gen. slide WU0124) **14** Xiamen,Fujian (gen. slide WU0123) **15** Xingshan, Hubei (gen. slide WU0126) **16** Jingzhou, Hubei (gen. slide WU0125) **17** Wuyishan, Fujian (gen. slide WU0121a).

#### Distribution.

Heilongjiang, Jilin, Liaoning, Inner Mongolia, Beijing, Hebei, Shandong, Henan, Shaanxi, Qinghai, Jiangsu, Shanghai, Zhejiang, Hubei, Jiangxi, Fujian, Taiwan, Guangdong, Guangxi; Russia (Far East), Korea, Japan.

#### Remarks.

*Monema melli* was described based on a single male from Guangdong, China. It differs from *Monema flavescens* by the smaller size and the shorter labial palpus. According to the male genitalia of the holotype examined and provided by Mr. Solovyev, it matches well with that of *Monema flavescens*. Thus we synonymize *Monema melli* with *Monema flavescens*. Solovyev and Witt (2009) treated *Monema flavescens* var. *nigrans* as a full species. However, the black form (var. *nigrans*) and the normal form (*flavescens*) are from the same population and the var. *nigrans* do not show any differences in the male or female genitalia with *flavescens*. Therefore we treat *Monema flavescens* var. *nigrans* merely as a dark form. In Guiyang 2.75% of the population of *flavescens* belonged to the black form; the black individuals can interbreed with the non black individuals and produce fertile offspring (Long et al. 2008).

### (a) Monema flavescens flavescens

Walker, 1855

http://species-id.net/wiki/Monema_flavescens_flavescens

Figs 5, 6 , 12 , 13, 14, 15, 16, 17 , 22, 23

Monema flavescens Walker, 1855: 1112, fig. 1c

#### Description.

Wing expanse 25–39 mm. The aedeagus is 1.6 times as long as valva, shorter than that in ssp. *rubriceps*. Frons yellow to yellowish red.

#### Specimens examined.

Heilongjiang, Dailing, 390m, 30 June-16 July 1962, Bai Jiuju (25♂), 4–9 July 1957 (5♂, gen. slide WU0180); Heilongjiang, Yichun, 9 July 1956 (2♂), 6 Sept. 1956 (1♂); Heilongjiang, Wuchang, Shengli Linchang, 10 July 1970 (3♂); Heilongjiang, Harbin, 17 July–17 Aug. 1936 (2♀), 81 Aug. 1940; Heilongjiang, Hulin 852 Farm, 10 July 1962, Chen Tailu (1♂); Liaoning, Qingyuan, 29–30 July 1954 (1♀8♂, gen. slide WU0179); Liaoning, XInjin, 1954 (2♂); Jilin, Manjiang, 19 June–27 July 1955 (4♀10♂); Jilin, Changbai Shan, 800m, 2–13 July 1982, Zhang Baolin (2♀3♂, gen. slide WU0178); Inner Mongolia, Ulanhot 15 July 1987 (1♂), 5 June 1957 (1♂, gen. slide WU0181); Hunan,Yongshun Shanmuhe, 600m, 3 Aug. 1988, Chen Yixin (1♂, gen. slide WU0117); Hunan, Andong, 20 May 1954 (1♂, gen. slide WU0118); Hunan, Guzhang Gaowangjie, 850m, 29 July 1988, Chen Yixin (1♂, gen. slide WU0120); Hunan, Hengshan, 22Aug. 1979 (1♂, gen. slide WU0119); Fujian, Wuyishan, Sangang, 3 Aug. 1979, Song Shimei (1♀, gen. slide WU0121); Fujian, Wuyishan, Tongmu, 26 July 1979, Song Shimei (1♂, gen. slide WU0121a); Fujian, Xiamen, 1♂, 25 June 1973, Zhang Baolin (1♂, gen. slide WU0123); Hubei, Shennongjia, 950–1640m, 18–24 July 1980, Yu Peiyu (8♂, gen. slide WU0124); Hubei, Jingzhou, July 1980 (6♂, gen. slide WU0125); Hubei, Xingshan Longmen River, 1350m, 16 June–17 July 1993, Yao Jian (9♂, gen. slide WU0126); Hubei, Zigui, Jiutouling, 100m, 12–13 June 1993, Yao Jian (3♂, gen. slide WU0127); Hubei, Xuanen, Fengshuiling 1200–1240m, 29 July 1989, Yang Longlong, Li Wei (1♂, gen. slide WU0128); Guangxi, Longsheng, 10–15 June 1980, Wang Linyao (4♂), 26 May 1963, Wang Chunguang (1♀, gen. slide WU0133, 134); Guangxi, Gualin Forestry Institute, 5 July 1981, Liang Xinqiang (1♂, gen. slide WU0145); Guangxi, Qinzhou, 15 Apr. 1980, Cai Rongquan (2♂, gen. slide WU0143); Guangxi, Jinxiu, 1100m, 10 May 1999, LI Wenzhu (1♂) (gen. slide WU0141); Zhejiang, Hangzhou, 1 Aug. 1973, Zhang Baolin (2♂); Zhejiang, Hangzhou, 4–21 June 1976, Chen Ruijin (1♀3♂); Zhejiang, Wenzhou, 1953, Liao Dingxi (1♂); Zhejiang, Zhoushan, 18 June 1936, O. Piel (1♂); Zhejiang, Tianmu shan, May-July 1936 (1♀3♂), 29 July 1972, Wang Ziqing (4♂), 21 July 1973, Zhang Baolin (5♂, gen. slide WU0146); Shaanxi, Zhouzhi, 1350m, 24 June 1999, Yao Jian (3♂, gen. slide WU0174, 175) Jiangxi, Lushan, 17–19 June 1974, Zhang Baolin (2♂) (gen. slide WU0146); Jiangxi, Guling, July 1935 (1♀1♂, gen. slide WU01149); Jiangxi, 27–28 May 1957, Yu Peiyu (1♀2♂, gen. slide WU0150); Shanghai, 11–26Aug.1932, O. Piel (16♀4♂, gen. slides L06056, 57), 14 June- 20 July 1933, A. Savio (4♂); Shanghai, Botanical Park, June 1974 (1♀); Jiangsu, Yangzhou, 15 May 1926 (1♂), 20 June 1974 (1♂, gen. slide WU0182); Jiangsu, Nanjing, 1–10 June 1957, Yu Peiyu (3♀) (gen. slide WU0183); Guangdong, Guangzhou, July 1931 (5♂); Guangdong, Guangzhou, Shipai, 17 Sept. 1958, Wang Linyao (1♂); Guangdong, Nanling, 21 July 2008, Chen Fuqiang (1♂, gen. slide WU0053a) Beijing, 3–31 May 1957, Yu Peiyu (6♀15♂, gen. slide WU0184); Beijing, Xishan, 1♂, Aug.1955; Beijing, Qinghe, 1♂, 13 Mar.1957 (1♂); Beijing, Tanzhesi, 15 Aug. 1951 (1♂); Beijing, Bada ling, 24 June 1957 (1♂); Beijing, Baihua shan, 4–16 July 1973, Liu Youqiao, Zhang Baolin (5♂, gen. slide WU0185); Beijing, Sanbu, 25 July 1964, (5♂), 21 July 1972, Zhang Baolin (1♀); Hebei, Changli, 15 June-8 July 1972 (1♂6♀), 21 June 1973 (2♀, gen. slide WU0186); Henan, Songxian, Baiyun shan, 1400m, 18–20 July 2003, Qiu Reng (2♂); Henan, Huixian Baligou, 700m, 12–15 July 2002 (1♀); Henan, Neixiang Baotianman, 12 July 1998, Shen Xiaocheng (1♂). **Black form:** Shanghai Datong Route, 28 July 1980 (1♂, gen. slide L06052); Shanghai Botanical Park, June 1974, Tian Lixin (1♀) (gen. slide L06053); Shanghai, July 1935 (2♂); Jilin, Manjiang, 9–31 July 1955 (1♀2♂, gen. slides L06059, L06060).

#### Distribution.

Mainland China; Russia (Far East), Korea, Japan.

### (b) Monema flavescens rubriceps

(Matsumura, 1931) stat. n.

http://species-id.net/wiki/Monema_flavescens_rubriceps

Fig. 10

Cnidocampa rubriceps Matsumura, 1931: 105. Type locality: Taiwan, China.
Monema rubriceps (Matsumura): Hering, 1931: 691.

#### Description.

Wing expanse 30–32mm. It differs from *Monema flavescens flavescens* Walker by the red frons. The aedeagus about twice as long as valva, longer than that of ssp. *flavescens*.

#### Specimens examined.

None. The image of the male genitalia of *Monema rubriceps* (Matsumura) was provided by Dr. Solovyev.

#### Distribution.

Taiwan.

#### Remarks.

*Cnidocampa rubriceps* is treated here as a subspecies of *Monema flavescens* because the male genitalia have the same structure, except for the aedeagus that is longer in ssp. *rubriceps* than in ssp. *flavescens*.

### Monema tanaognatha

Wu & Pan, sp. n.

urn:lsid:zoobank.org:act:AAF50A42-14EA-45E5-AAD3-0F2E027119A8

http://species-id.net/wiki/Monema_tanaognatha

Figs 1, 2 , 7 , 18

#### Description.

Wing expanse 28–33mm. Labial palpus yellowish brown, tip black. Face yellow to pale red. Head and thorax yellow. Abdomen yellowish brown. Ground colour of forewing yellow, with two dark concave fasciae from apex to 1/3 and 2/3 of inner margin, distal part of forewing, bordered by proximal fascia, brown. Hindwing yellow to yellowish brown.

Male genitalia: tegumen narrow; uncus narrow and long, ventrally with short process on apex; gnathos narrow and very long; juxta U-shaped, each lateral bar with distal tuft of long spines; valva elongate, with a strong short apically saccular process; saccus long and relatively narrower than that of *flavescens*; aedeagus slightly longer than valva, narrow and straight.

Female genitalia: posterior apophysis long, anterior apophysis very short; sclerotized exterior flap at posterior margin of ostium smaller, bearing minute hair; ductus bursae very long, basal half narrow and straight, apical half wider and spiraled; corpus bursae ovate, large; a pair of signa trigonal, bearing spines.

**Figures 18–21.**
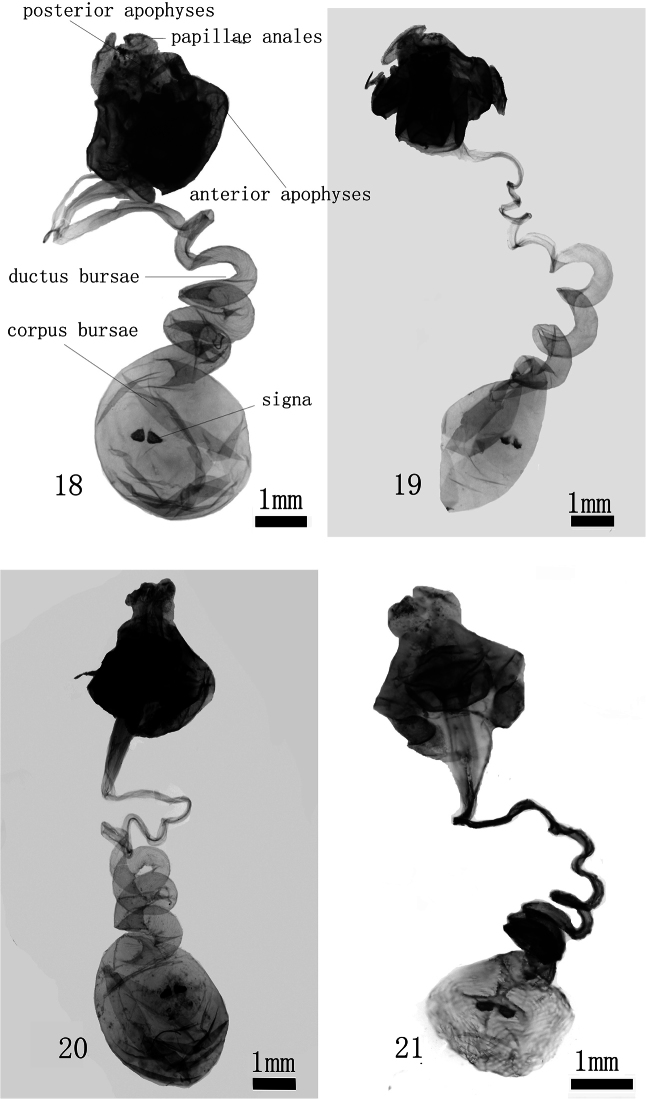
Female genitalia. **18** *Monema tanaognatha* Wu & Pan sp. n. (gen. slide WU0156) **19** *Monema meyi* Solovyev &Witt (gen. slide WU0147) **20** *Monema flavescens flavescens* Walker (gen. slide WU0121) **21** *Monema flavescens flavescens* Walker (Black form (gen. slide L06053).

**Figures 22–23.**
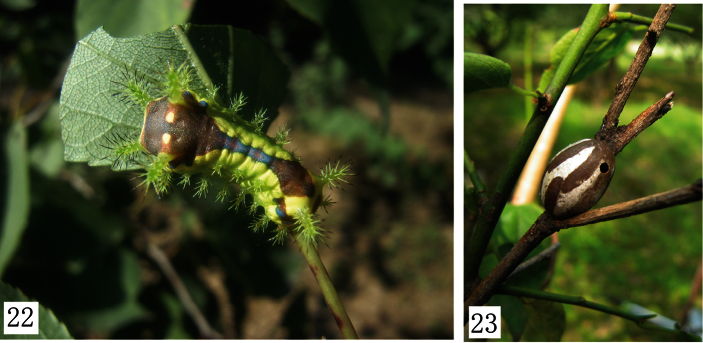
Larva and cocoon of *Monema flavescens flavescens* Walker. **22** larva **23** cocoon.

#### Type material.

Holotype: male, Kunming, Yunnan, 16 May 1980, Song Shimei (gen. slide WU0156). Paratypes: 1♀♂, same data as holotype (gen. slide WU0157); Xuanwei, Yunna, 1890m, 1♂, 25 June 1979 (gen. slide WU0152). Shaanxi, Fuping, 900–950m, 23–24 July 1998, Yuan Decheng, Yao jian, Zhang Youwei (5♂,gen. slide WU0172); Shaanxi, Liuba Miaotaizi, 1350m, 21 July 1998, Yao jian (1♂); Shaanxi, Taibai Huangbaiyuan, 1350m, 14 July 1980, Zhang Baolin (4♂, gen. slide WU0173); Shaanxi, Ningshan Huoditang, 1580–1650m, 27 July 1998, Yao Jian (4♂, gen. slides WU0171, WU0176); Gansu, Wenxian Tielou, 1450m, 1999 July 24, Yao jian, Wang Hongjian, Zhu Chaodong (7♂, gen. slide WU0164); Gansu, Kangxian, Qinghe linchang, 1400–2250m, 15 July 1998, Yao Jian (3♂), 7–9 July 1999, Yao jian (9♂, gen. slide WU0162, 163); Gansu, Kangxian, Baiyun shan, 1250–1750m, 12 July 1998, Yao Jian(3♂) (gen. slide WU0165); Gansu, Diechang, 1800m, 7 July 1998, Yao Jian (4♂, gen. slide WU0166); Gansu, Zhouqu, Shantan Linchang, 2400m, 15 July 1999, Wang Hongjian (4♂, gen. slide WU0167, 168); Sichua, Emei shan, 800–1000m, 21 June-25 July 1957, Huang Keren, Zhu fuxing, Lu Youcai (7♂, gen. slides WU0110, WU0135); Sichua, Dujiang Yan, Qingcheng Shan, 700–1000m, 3–4 June 1979, Gao Ping, Shang Jinwen (2♂) (gen. slides WU0112, WU0113); Hubei, Lichuan, Xingdou Shan, 800m, 21–23 July 1989, Li Wei (2♂, gen. slide WU0130); Guangxi, Miaoer shan, Jiuniuchang, 1150m, 7 July 1985, Fang Chenglai (2♂, gen. slide WU0132).

#### Distribution.

Shaanxi, Gansu, Hubei, Guangxi, Sichuan, Yunnan.

#### Remarks.

The species is similar externally to *Monema flavescens*, but is well distinguished by male genitalia: gnathos is narrowing and very long; juxta is long and ending in a tuft of long spines at each side. In female genitalia, base of ductus bursae of the new species is narrow and membranous, while that of *Monema flavescens* is sclerotized.

#### Etymology.

The name is derived from Greek “Tanaos” (=Long) and “gnathos” (Greek for jaw), corresponding to the long gnathos in the male genitalia.

### Monema meyi

Solovyev & Witt, 2009, new record to China

http://species-id.net/wiki/Monema_meyi

Figs 3 , 9 , 19

Monema meyi Solovyev & Witt, 2009: 108–109. Type locality: Vietnam (ZMHB).

#### Description.

Wing expanse 35-38 mm in male, 36-42 mm in female. The species is similar externally to *Monema flavescens*, but well discriminated by male genitalia: saccular process divided apically, juxta with lateral row of elongate spines, very broad saccus, S-shaped aedeagus with long and strong apical spur.

Female genitalia: posterior apophysis long, anterior apophysis very short; sclerotized exterior flap at posterior margin of ostium large and elongate, bearing minute hair; ductus bursae very long, basal 1/3 narrow and straight, apical 2/3 wider and spiralled; corpus bursae ovate, large; a pair of signa trigonal, bearing spines.

#### Specimens examined.

Hunan, Sangzhi, Baxixiang, 370m, 13 July 2009, Chen Fuqiang (1♀) (gen. slide WU0052); Guangdong, Chebaling (2♀8♂, gen. slides WU0053, WU0054, WU0054a); Sichuan, Emei Shan, 800-1000m, 21 June-25 July 1957, Huang Keren, Zhu Fuxing, Lu Youcai (1♀8♂, gen. slide WU0111); Sichuan, 21-24 July 1974 (1♂, gen. slide WU0137); Guizhou, Jiangkou, Fanjing Shan, 500m, 11 July 1988, Li Wei (1♀2♂, gen. slides WU0115, WU0116); Fujian, Wuyishan, 14 June 1982, Zhang Baolin (1♂, gen. slide WU0177); Jiangle, Longqi Shan, 18Aug. 1991, Song Shimei (1♂, gen. slide WU0121); Hubei, Xuanen, Fengshuiling, 1200-1240m, 25 July 1989, Yang Longlong, Li Wei (1♂, gen. slide WU0129); Hubei, Lichuan, Xingdou Shan, 800m, 21-31 July 1989, Li Wei (3♂, gen. slide WU0138); Hubei, Hefeng, Fengshuiling, 1240m, 29 July 1989, Li Wei(1♂, gen. slide WU0131); Guangxi, Jinxiu, Shengtang Shan, 900m, 17 May 1999, Li Wenzhu (1♂, gen. slide WU0140); Guangxi, Jinxiu Luoxiang, 200-400m, 15-16 May 1999, Han Hongxiang (5♂, gen. slide WU0142); Guangxi, Shangsi Hongqi Linchang, 250m, 28 May 1999, Yuan Decheng (1♂); Dayu, 16 Aug. 1985, Wang Ziqing (1♀); Jiangxi, Deyu Neiliang, 23 Aug. 1985 (1♂, gen. slide WU0150); Jiangxi, Yifengyuan, 2 June 1959 (1♂, gen. slide WU0151); Hainan, Wuzhi Shan, 25 Apr. 1984, Gu Maobin (1♀, gen. slide WU0147); Yunnan, Menghai, 1200m, 18 July 1958, Wang Shuyong (1♂, gen. slide WU0153); Yunnan, Binchuan, Aug. 1959 (2♂, gen. slide WU0154); Yunnan, Weixi, 2320m, 6 July 1979 (1♂, gen. slide WU0155).

#### Distribution.

Hubei, Hunan, Fujian, Jiangxi, Guangdong, Hainan, Guangxi, Sichuan, Guizhou, Yunnan; Vietnam.

#### Remarks.

This species, newly recorded in China, was described based on two males from Vietnam (Solovyev and Witt 2009). This is the first report and description of the female. The sclerotized exterior flap at posterior margin of ostium is large and elongate compared to that found in *Monema flavescens* and *Monema tanaognatha*.

### Monema coralina

Dudgeon, 1895, new record to China

http://species-id.net/wiki/Monema_coralina

Figs 4 , 8

Monema coralina Dudgeon, 1895: 290. Type locality: Bhutan.

#### Description.

Wing expanse 30-35mm. The mostly reddish wings are diagnostic. In the male genitalia, the uncus lacks the ventrally apical process.

#### Specimens examined.

Yunnan, Xinshuangbanna, 700m, 4-15 Apr. 1993, Yang Longlong (4♂, gen. slide L06051); Xizang, Motuo, 1080m, 22 July 2006, Chen Fuqiang (1♀).

#### Distribution.

Yunnan (Xinshuangbanna), Xizang (Motuo); Nepal, Bhutan.

#### Remarks.

The abdomen of the female from Xizang is missing. The species is reported for the first time in China.

**Figure 24.**
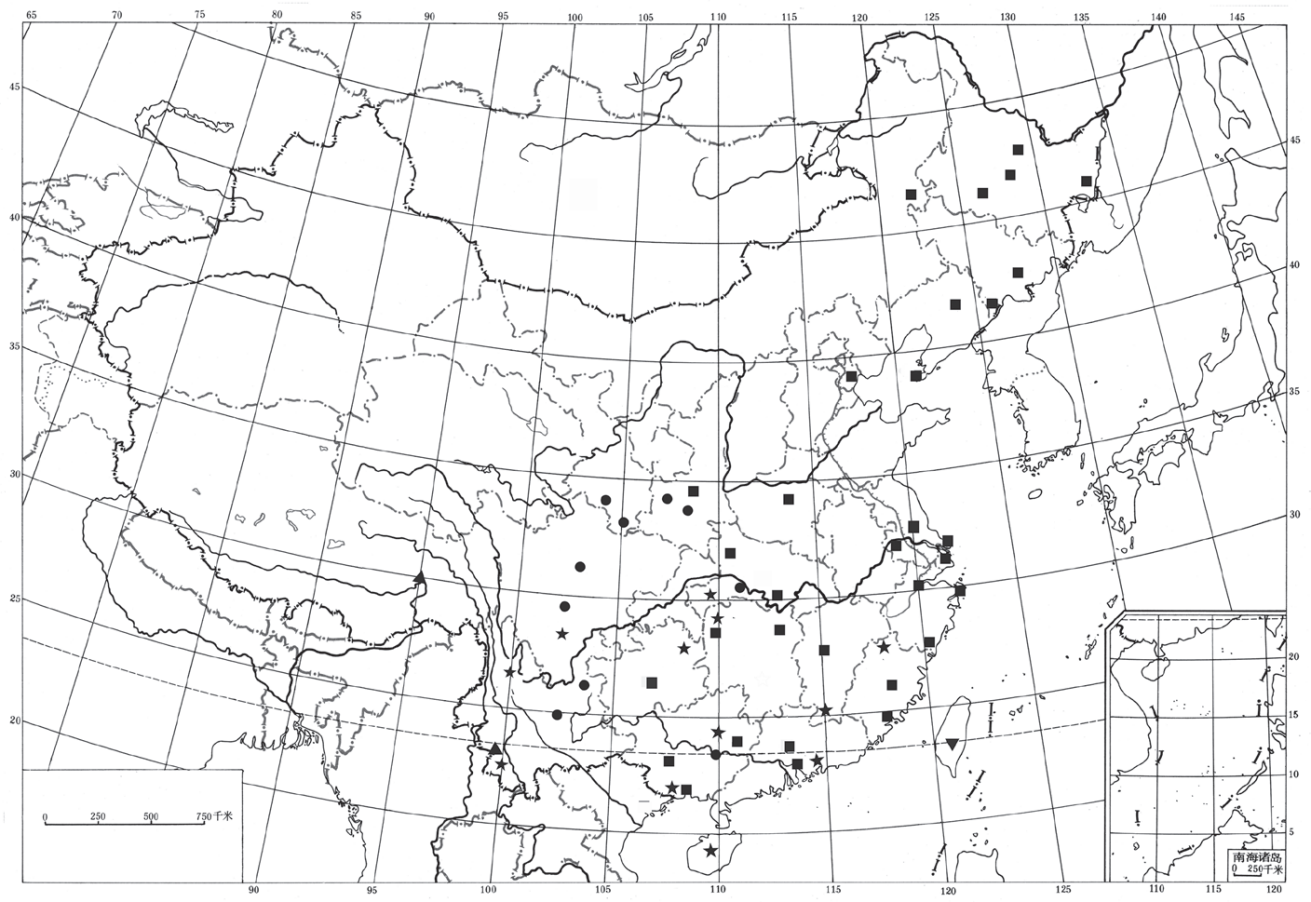
Distribution of *Monema* in China. ● *Monema tanaognatha* Wu & Pan; ▲ *Monema coralina* Dudgeon; ★ *Monema meyi* Solovyev & Witt; ■ *Monema flavescens flavescens* Walker; ▼ *Monema flavescens rubriceps* (Matsumura).

## Supplementary Material

XML Treatment for Monema

XML Treatment for Monema flavescens

XML Treatment for Monema flavescens flavescens

XML Treatment for Monema flavescens rubriceps

XML Treatment for Monema tanaognatha

XML Treatment for Monema meyi

XML Treatment for Monema coralina
